# Chestnut polysaccharide rescues the damaged spermatogenesis process of asthenozoospermia-model mice by upregulating the level of palmitic acid

**DOI:** 10.3389/fendo.2023.1222635

**Published:** 2023-07-05

**Authors:** Baoquan Han, Jiachen Guo, Bo Zhou, Chunxiao Li, Tian Qiao, Lei Hua, Yinuo Jiang, Zihang Mai, Shuai Yu, Yu Tian, Xiaoyuan Zhang, Dongliang Lu, Bin Wang, Zhongyi Sun, Lan Li

**Affiliations:** ^1^ College of Life Sciences, Qingdao Agricultural University, Qingdao, China; ^2^ Department of Urology, Shenzhen University General Hospital, Shenzhen, China; ^3^ Department of Urology, Daping Hospital, Army Medical University, Chongqing, China

**Keywords:** chestnut polysaccharide, palmitic acid, asthenozoospermia, spermatogenesis, hydrogen carbonate

## Abstract

**Introduction:**

In recent years, the quality of male semen has been decreasing, and the number of male infertilities caused by asthenozoospermia is increasing year by year, and the diagnosis and treatment of patients with asthenozoospermia are gradually receiving the attention of the whole society. Due to the unknown etiology and complex pathogenesis, there is no specific treatment for asthenozoospermia. Our previous study found that the administration of chestnut polysaccharide could alter the intestinal microbiota and thus improve the testicular microenvironment, and rescue the impaired spermatogenesis process by enhancing the expression of reproduction-related genes, but its exact metabolome-related repairment mechanism of chestnut polysaccharide is still unclear.

**Methods and results:**

In this study, we studied the blood metabolomic changes of busulfan-induced asthenozoospermia-model mice before and after oral administration of chestnut polysaccharide with the help of metabolome, and screened two key differential metabolites (hydrogen carbonate and palmitic acid) from the set of metabolomic changes; we then analyzed the correlation between several metabolites and between different metabolites and intestinal flora by correlation analysis, and found that palmitic acid in the blood serum of mice after oral administration of chestnut polysaccharide had different degrees of correlation with various metabolites, and palmitic acid level had a significant positive correlation with the abundance of *Verrucomicrobia*; finally, we verified the role of palmitic acid in rescuing the damaged spermatogenesis process by using asthenozoospermia-model mice, and screened the key target gene for palmitic acid to play the rescuing effect by integrating the analysis of multiple databases.

**Discussion:**

In conclusion, this study found that chestnut polysaccharide rescued the damaged spermatogenesis in asthenozoospermia-model mice by upregulating palmitic acid level, which will provide theoretical basis and technical support for the use of chestnut polysaccharide in the treatment of asthenozoospermia.

## Highlights

1. Hydrogen carbonate may be significantly involved with the chestnut polysaccharide rescue process.

2. Upregulated palmitic acid is important for rescuing the damaged spermatogenesis process by using chestnut polysaccharide.

3. Palmitic acid may rescue the damaged spermatogenesis process of asthenozoospermia-model mice via enhancing *PPARA* expression.

## Introduction

Currently, infertility affects approximately 60 to 80 million couples worldwide at a rate of 15% (1), and the World Health Organization (WHO) ranks infertility as the third most common disease after oncology and cardiovascular disease, with male factors accounting for approximately 50% of infertility (2). In recent years, the quality of male semen is decreasing, the number of male infertilities is increasing year by year, and the issue of male reproductive health is of concern to the whole society. From the perspective of semen quality, male infertility is usually a condition caused by reduced semen quality, with the most common clinical manifestations being low sperm count (oligospermia), poor sperm motility (weak spermatozoa) and abnormal sperm shape (abnormal spermatozoa). According to the latest WHO clinical guidelines, patients with a sperm progressive motility (PR) <32% in semen are diagnosed with weak spermatozoa, which is characterized by reduced sperm motility and decreased sperm motility (3). In 2003, Curi et al. reported that 80% of male infertility was associated with impaired sperm motility and 20% of male infertility was directly related to low sperm motility (4), this study fully demonstrates that weak spermatozoa are an important cause of the occurrence of male infertility, and considering that normal sperm motility is necessary for the completion of fertilization, and that male infertility patients with weak spermatozoa have significantly reduced sperm motility as a clinical manifestation, sperm motility is essential for maintaining normal male fertility.

Due to the unknown etiology and complex pathogenesis, there is no specific treatment for weak spermatozoa, and researchers have been investigating the use of antioxidant therapy or lifestyle changes to improve sperm quality. Studies have shown that lifestyle changes can significantly improve semen quality and sperm motility, such as reducing smoking and alcohol consumption can improve sperm motility (5). In addition, studies have found that obese patients can improve testicular function and enhance sperm motility through weight loss and regular exercise. Also, increasing the number of intercourse and ejaculation can improve sperm motility (6). Whereas antioxidant therapy is widely used by clinicians to improve sperm quality (7), it is now commonly used clinically through supplementation with carnitine (8), vitamin E (9), selenium (10), or acetylcysteine (11) to improve semen quality. However, the therapeutic potential of antioxidants remains controversial because of insufficient clinical sample sizes (12). In addition, some therapeutic approaches, including L-carnitine, are inefficient, costly, or have potential side effects when used, and there is still an urgent need for efficient, low-cost, and non-toxic alternative therapies for the effective treatment of weak spermatozoa.

Chestnut (*Castanea mollissima Blume*) is a plant of the family Crustaceae, which is widely grown in most parts of China and is an ingredient of traditional Chinese medicine. Chestnut is rich in nutrients such as starch, soluble sugar, crude fiber, protein, amino acids, and minerals (13). In recent years, polysaccharides have received increasing attention due to their multiple biological activities such as antioxidant, anti-inflammatory, immunostimulatory, anti-proliferative and anti-cancer (14, 15). Chestnut polysaccharides are the main components in chestnuts and consist of monosaccharides in the α or β conformation, linked by glycosidic bonds (16). Chestnut polysaccharides (CPs) include many monosaccharides such as glucose, rhamnose, arabinose, galactose, xylose, mannose, and fructose. CPs have been shown to have anticancer activity (17) and anti-fatigue effect. In addition, chestnut extract was found to improve the tolerance and survival of lactic acid bacteria in the gastrointestinal tract (18), and a recent study (19) showed that the addition of chestnut starch to the diet of mice altered the ratio of cecum-associated microorganisms and associated carbohydrate metabolites (e.g., acetic acid). In addition, chestnut starch induced changes in the expression of several genes in cecum epithelial cells, including those involved in energy production, cell cycle and cell junctions (20). This also confirmed that the components of chestnut starch can alter the gut microbiota and affect the expression of microbial metabolites and host genes, providing an important theoretical basis for the development of this project. Our team discovered that chestnut polysaccharide could enhance the expression of reproduction-related genes (STRA8, DAZL, SYCP1, SYCP3 and TNP1) to rescue the impaired spermatogenesis process (21). Moreover, another study confirmed that CPs can restore the impaired spermatogenesis process by adjusting the gut microbiota and intestinal structure (19), which also laid an important foundation for the subsequent research work of this project.

Palmitic acid (PA) is a major saturated fatty acid commonly found in sperm (22). In 2008, a study showed that PA levels were higher in semen samples from patients with asthenozoospermia than in the normal population (23). in addition, Kiernan et al. found that the addition of PA to an extender improved sperm quality in bulls (24). A recent clinical study found a positive correlation between high PA intake and the incidence of asthenozoospermia (25), while Andersen et al. also found a positive correlation between PA in sperm and total sperm count, further confirming the importance of PA for sperm production (22). Recent studies reported that PA has an important effect on maintaining linear motility and viability of porcine spermatozoa (26), which provides an important theoretical basis for conducting this study.

Metabolomics is an emerging histology that emerged after genomics, transcriptomics, and proteomics with the goal of quantitatively describing metabolite changes in organisms. This histological approach can reflect events downstream of gene expression and is closer to the actual phenotype than proteomics and genomics (27). Our previous study found that the administration of chestnut polysaccharide could alter the intestinal microbiota and thus improve the testicular microenvironment, and rescue the impaired spermatogenesis process by enhancing the expression of reproduction-related genes, but its exact metabolome-related repairmen mechanism of chestnut polysaccharide is still unclear. In this study, we studied the blood metabolomic changes of busulfan-induced asthenozoospermia-model mice before and after oral administration of chestnut polysaccharide with the help of metabolome, which will provide a more in-depth dissection of the molecular mechanism of chestnut polysaccharide rescuing the impaired spermatogenesis process and provide a new direction for the clinical prevention and treatment of asthenozoospermia.

## Materials and methods

### The design of this study

**Figure 1:**
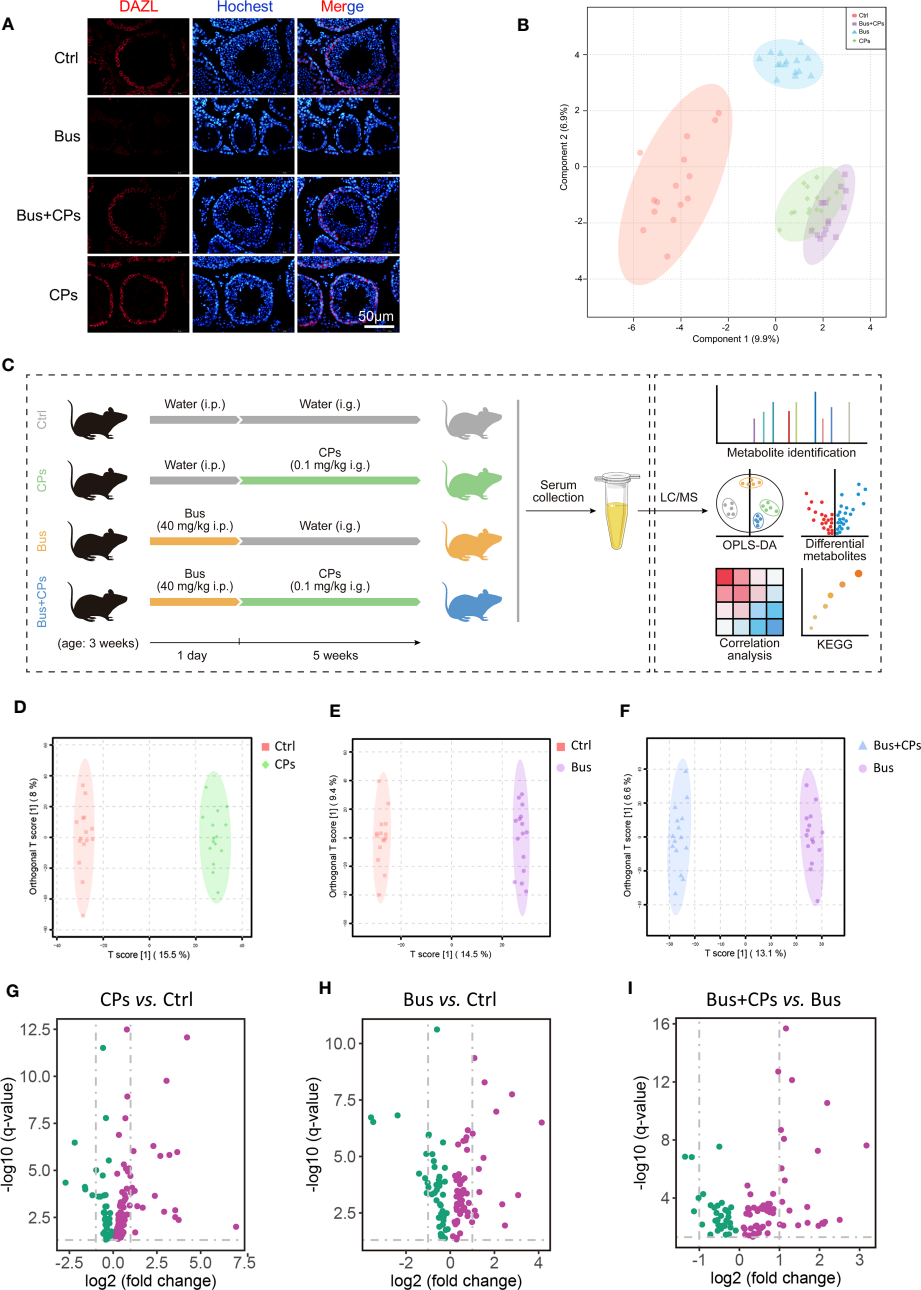
Study design and CPs-produced changes in metabolic features. **(A)** Histopathology photos of DAZL staining of mice testes; **(B)** the beta diversity analysis between the different groups by Principal Coordinates Analysis (PCoA); **(C)** the whole design of the study; **(D-F)**. the Orthogonal Partial least squares discriminant analysis (OPLS-DA) of diverse groups; **(G-I)**. the volcano map of differential expressed metabolites from different comparison groups.

Based on the existing research reports and experimental validation (**Figure 1A**), we conducted the following design for this study as displayed in **Figure 1C**: we set up a total of four experimental groups, including control group (Ctrl), oral administration of chestnut polysaccharide group (CPs), asthenozoospermia-model group (Bus) and chestnut polysaccharide rescue group (Bus+CPs). The treatment was started from 3 weeks of age, and subsequently, after one spermatogenic cycle (5 weeks), blood serum samples from different treatment groups were collected for metabolome assay analysis, and the metabolite composition and differential metabolite functions among different groups were analyzed in detail by various methods, so as to determine the effects of CPs on the serum metabolome of asthenozoospermia-model mice and the key metabolites that play important roles in rescuing damaged spermatogenesis process.

### Breeding environment of mice

Male ICR mice were purchased from Vital River Laboratory Animal Technology Co., Ltd (Beijing, China). The mice were kept in a house with a 12-hour cycle of light and12-hour cycle of dark and a constant temperature (22–23°C) and had free access to food and water during the experimental phase. The Animal Care and Ethics Committee of Qingdao Agricultural University approved the study, which was conducted in accordance with the National Institutes of Health guidelines for the care and use of laboratory animals (NIH Publications no. 8023).

### Information of CPs and busulfan

The CPs used in this study were brought from Wo Te Lai Si bio-technology co., Ltd (Lan Zhou, China) and busulfan (B2635, Germany) was brought from Sigma-Aldrich company.

### Treatment of mice

Busulfan was used to establish the asthenozoospermia model of male sterility. Different treatment groups (10 mice per group) are as described in “The design of this study”. The asthenozoospermia-model mice were treated with busulfan at the concentration of 40 mg per kg body weight. Fresh CPs solutions were prepared daily and 0.1ml of CPs solution were given orally per day at the rate of 0.1mg per kg body weight every mouse.

### Collection of samples

After five-week treatment, mice were slaughtered in accordance with animal welfare requirements, and the tissues were collected for further analysis. Mouse blood samples are collected as follows: grasp the skin of the animal’s neck with the left hand, take the lateral position to press it lightly on the experimental table, the left thumb and forefinger press the animal’s eye skin to the back of the neck as much as possible, so that the animal’s eye is filled with blood and protrudes, and the eyeball is removed with curved forceps, and the mouse is inverted with the head downward to make the blood flow out. After that, the blood serum was extracted and stored at -80°C until use.

### Tissue immunofluorescence

Collected testes were fixed in 4% paraformaldehyde and kept in a refrigerator at 4°C overnight, then subsequently stored in different concentrations of dehydrating solutions. The dehydrated testicular samples were then embedded in paraffin and the resulting paraffin blocks were sectioned at 5 μm thickness following standard histological procedures. Sections were deparaffinized and hydrated in xylene and ethanol. Antigen retrieval was performed using citrate solution. Sections were then blocked with blocking buffer [3% bovine serum albumin (BSA, Solarbio, A8020, China), 10% normal goat serum in TBS buffer] at room temperature for 30 min. Each section was incubated with primary antibodies-DAZL (Abcam, ab215718, USA) and secondary antibodies (Beyotime, A0516, China) then sections were imaged under an Olympus fluorescence microscope (Olympus, BX51, Tokyo, Japan).

### Metabolites extraction and UHPLC-MS/MS analysis

Blood serum samples from treated mice were collected, placed in Eppendorf tubes per 100 μL, and resuspended with prechilled 80% methanol in a well vortex. Dilute part of the supernatant with LC-MS grade water to a final concentration containing 53% methanol. Samples are then transferred to a new Eppendorf tube and centrifuged at 15,000 g, 4°C for 20 min. Finally, the supernatant is injected into the LC-MS/MS system for analysis. UHPLC-MS/MS analysis was performed using the Vanquish UHPLC System (Thermo Fisher Scientific Technologies) and the Orbitrap Q ExactiveTM high-frequency mass spectrometer (Thermo Fisher Scientific Inc.) at Novogene Co., Ltd. (Beijing, China). The Q ExactiveTM HF mass spectrometer operates in positive/negative polarity mode with a spray voltage of 3.5 kV, a capillary temperature of 320°C, a sheath flow rate of 35 arb, an auxiliary gas flow rate of 10 arb, an S-lens RF level of 60, and an auxiliary gas heater temperature of 350°C.

### Metabolite profiling from different samples

Raw data files generated by UHPLC-MS/MS were processed using the Compound Discoverer 3.1 (Thermo Fisher Scientific, USA) to perform peak alignment, peak picking, and quantitation for each metabolite. Subsequently, peak intensities were normalized to the total spectral intensity. The normalized data was used to predict the molecular formula based on additive ions, molecular ion peaks, and fragment ions. Peaks were then matched with the mzCloud, mzVault, and MassList databases to obtain accurate qualitative and relative quantitative results. Statistical analyses were performed using the statistical software R (v.3.4.3), Python (v.2.7.6) and CentOS (v.6.6), When data were not normally distributed, normal transformations were attempted using the area normalization method. These metabolites were annotated using the KEGG database, HMDB database and LIPIDMaps database. Orthogonal Partial least squares discriminant analysis (OPLS-DA) were performed using metaX (flexible and comprehensive software for processing metabolomics data). We used univariate analysis (t-test) to calculate the statistical significance (p-value). Metabolites with VIP >1, *p*value *<*0.05, and log2 (fold change) ≥0 or log2 (fold change) ≤0 were considered to be differential metabolites. Volcano plots were used to filter metabolites of interest which were based on log2 (fold change) and -log10 (*p* value) of metabolites with ggplot2 in R language. For clustering heat maps, the data were normalized using z-scores of the intensity areas of differential metabolites and were plotted using the pheatmap package in R language. Correlation between differential metabolites were analyzed by cor () in R language (Pearson method). Statistically significant correlations between differential metabolites were calculated by cor.mtest () in R language. *p* value <0.05 was considered to be statistically significant and correlation plots were plotted using the corrplot package in R language. Functions of these metabolites and metabolic pathways were studied using the SMPDB database. Metabolic pathways enrichment of differential metabolites was performed; when the ratio was satisfied by x/n > y/N, the metabolic pathway was considered to be enriched, when the P-value of the metabolic pathway <0.05, the pathway was considered as significantly enriched.

### Prediction of target genes of candidate metabolite and expression profiling of target genes in different databases

Upon confirming the candidate metabolite, we predicted target genes of candidate metabolite using the Swiss Target-Prediction webtool (http://swisstargetprediction.ch/) and STITCH Database (http://stitch.embl.de/cgi/), then candidate genes were further screened by sorting according to the probability value and intersecting from two gene sets. After that, we conducted expression profiling of target gene from The Human Protein Atlas (https://www.proteinatlas.org/) to further explore its role.

### Detection of the promoting effects of the candidate metabolite in rescuing damaged spermatogenesis process *in vivo*

In this part of the experiment, asthenozoospermia-model mice were prepared with the aid of busulfan, with reference to existing literature (28, 29). Three-week-old male mice were used as experimental subjects, and a control group (Ctrl), a busulfan and metabolite co-injection group (Bus+PA), and a busulfan injection group (Bus) were set up, with six mice in each group. The candidate metabolite intraperitoneal injection concentration was calculated based on the concentrations screened in the preliminary experiment. After one spermatogenic cycle, testicular tissues of different treatment groups were collected and testicular coefficients were analyzed; sperm quality of mice in different treatment groups were also statistically analyzed through CASA to verify the effect of metabolite on sperm motility.

### Correlation analysis of 16S rDNA and metabolite profiling from blood serum

Based on our previous report (19), after completing metabolomics analyses of the blood serum, the Phylum that were significantly different after 16S rDNA profiling were correlated with the metabolites that were significantly different from the metabolite profiling based on Pearson’s correlation coefficient. Heat maps were drawn to measure the degree of association between species diversity and metabolites in environmental samples.

### Code availability

Analysis scripts employing these packages (and associated usage notes) are available from the authors upon request.

### Data availability

The microbiota raw sequencing data generated in this study have been uploaded to the Genome Sequence Archive (GSA) with the accession number CRA004367 that are publicly accessible at https://ngdc.cncb.ac.cn/gsa. Metabolomics data employed in this study are available from the authors upon request.

### Statistical analysis

All experiments were repeated at least 3 times and results were expressed as the mean ± SEM. SPSS software one-way analysis of variance (ANOVA) following by LSD multiple comparison test was used for data analysis and we defined *p* < 0.05 as a significant difference.

## Results

### Chestnut polysaccharide produced significant changes on the metabolome of asthenozoospermia-model mice

Consistent with our previous study (19), busulfan could significantly reduce the germ cells in seminiferous tubes, and CPs treatment effectively restored busulfan-impaired spermatogenesis, as evidenced by the increased number of germ cells in the Bus+Cps group mice (**Figure 1A**). After completing the UHPLC-MS/MS analysis, we firstly conducted the PCoA analysis of different samples and the results displayed that the spatial distribution of the Bus+CPs group and the CPs group was more similar which means similar components, while the Bus+CPs group was closer to the Ctrl group than the Bus group, indicating that CPs had a certain rescue effect on busulfan-induced spermatogenesis disorders (**Figure 1B**). Subsequently, the OPLS-DA analysis results (**Figure 1D-F**) further validated the influence of CPs or busulfan on metabolome components, especially for the asthenozoospermia-model mice (**Figure 1F**). In addition, the volcano plots (**Figure 1G-I**) were used to further show the metabolites differences between different groups. Based on these results, there were 38 significantly downregulated metabolites and 76 significantly upregulated metabolites in CPs vs Ctrl comparison; 46 downregulated metabolites and 61 upregulated metabolites in Bus vs Ctrl comparison; 32 downregulated metabolites and 64 upregulated metabolites in Bus+CPs vs Bus comparison. Above results demonstrated that CPs produced significant changes on the metabolome of asthenozoospermia-model mice, which further verified the important role of CPs-induced metabolome change in rescuing damaged spermatogenesis process.

**Figure 2:**
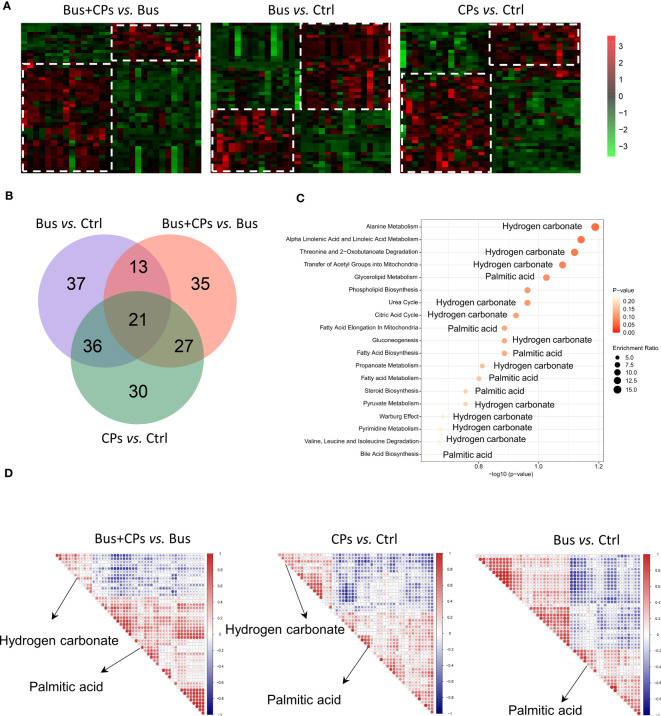
The candidate metabolites screening. **(A)** the expression heatmap of differential expressed metabolites from diverse comparison groups; **(B)** the cross-comparison analysis of the differential metabolites in the three comparison groups; **(C)** the enrichment analysis of the core 21 differential metabolites of the four comparison groups using the SMPDB database; **(D)** the correlation analysis of the top 50 differential metabolites in the three comparative groups.

Besides, the overall display of metabolites from three comparison showed large differences (**Figure 2A**), especially in Bus+CPs vs Bus comparison. It was found that after CPs gavage, more metabolites in asthenozoospermia-model group mice increased significantly than other two comparisons. Nevertheless, the Bus group had more significantly decreased metabolites than other two comparisons, which indicated that busulfan may severely damaged the spermatogenesis process by downregulating some important metabolites in mice. Also, it was detected that there was a significant different metabolome between the CPs and Ctrl groups, which suggests that CPs may have other potential effects in other biological process.

### Hydrogen carbonate may be significantly involved with the chestnut polysaccharide-mediated rescue process

**Figure 3:**
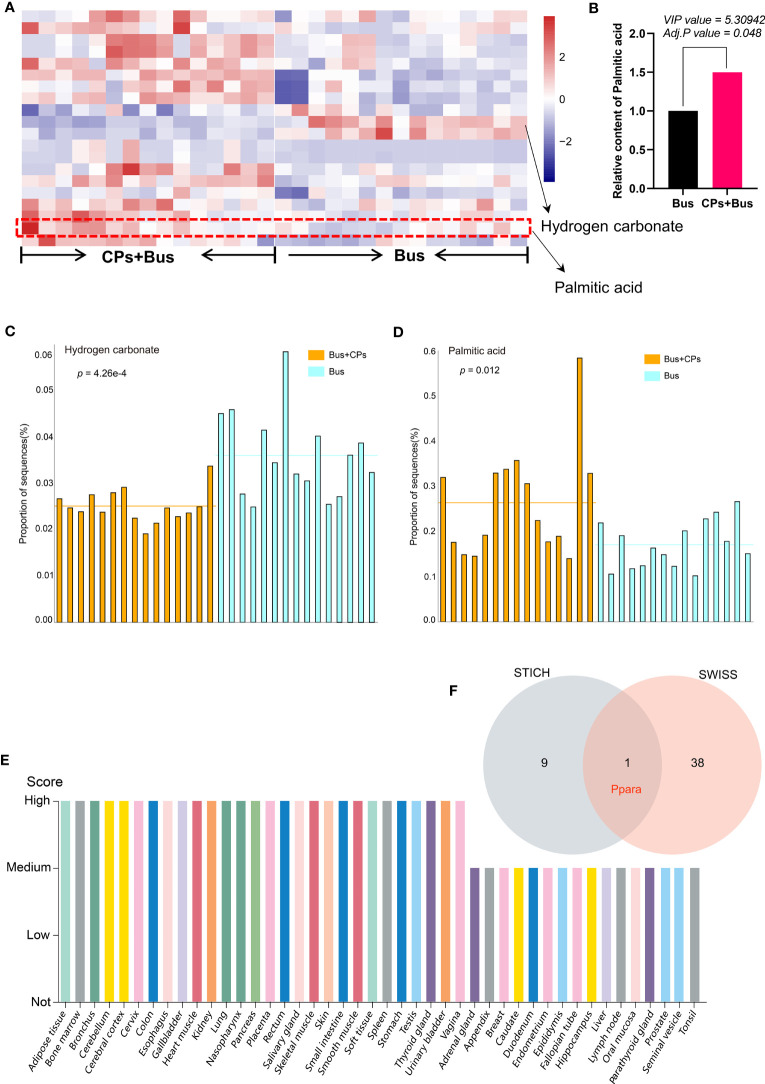
The expression display of key differential metabolites and screening of candidate target gene. **(A)** the heat map presentation of partial differential metabolites of the different groups in each sample; **(B)** the quantitative analysis of the palmitic acid from different groups; **(C, D)**. the STAMP analysis of Hydrogen carbonate and palmitic acid difference between groups; **(E)** the expression profiling of PPARA gene from The Human Protein Atlas; **(F)** the prediction and screening of target gene using different tools.

After confirming the effect of CPs on the metabolic composition of asthenozoospermia-model mice, we subsequently conducted a more in-depth study of the differential metabolites in different groups. Through cross-comparison analysis of the differential metabolites in the three comparison groups, we screened and obtained 21 differential metabolites that coexisted in the three comparison groups (**Figure 2B**), and with the help of functional enrichment analysis of the 21 differential metabolites, we found that among these core 21 differential metabolites, hydrogen carbonate and palmitic acid play an important role. We then correlated the top 50 differential metabolites in the three comparison groups and found that hydrogen carbonate and palmitic acid were also among the top 50 differential metabolites in the chestnut polysaccharide group, whereas hydrogen carbonate was not found among the top 50 differential metabolites in the model mice, which also demonstrated that oral administration of chestnut polysaccharide affected the hydrogen carbonate content in mice (**Figure 2C**). Subsequently, we performed heat map analysis (**Figure 3A**) and Stamp analysis (**Figure 3C**) on the metabolite composition of mice in the salvage and asthenozoospermia-model mice, and found that the hydrogen carbonate content of mice in the asthenozoospermia-model mice was significantly down-regulated after feeding chestnut polysaccharide, which suggest hydrogen carbonate may be significantly involved with the chestnut polysaccharide-mediated rescue process and also provides an important reference for the subsequent related studies.

### Upregulated palmitic acid is important for rescuing the damaged spermatogenesis process by using chestnut polysaccharide

In addition to the preliminary confirmation of the important role of hydrogen carbonate in the salvage of damaged spermatogenesis by chestnut polysaccharide, we found that palmitic acid also produced significant changes in the metabolome of chestnut polysaccharide-salvaged mice (**Figure 2B**), and the functional enrichment analysis revealed that palmitic acid plays an important role in biological processes such as “Glycerolipid metabolism”, “Fatty Acid metabolism”, “Fatty Acid Biosynthesis” and “Steroid Biosynthesis” (**Figure 2C**). Similarly, the correlation analysis of the top 50 differential metabolites in the three comparative groups showed that palmitic acid was included in the top 50 differential metabolites in all three comparisons, which is a preliminary evidence that the administration of chestnut polysaccharides can affect the spermatogenesis process by affecting palmitic acid content (**Figure 2D**). Subsequent heat map analysis (**Figures 3A, B**) and Stamp analysis (**Figure 3D**) revealed that the palmitic acid content was significantly up-regulated in the asthenozoospermia-model mice after feeding chestnut polysaccharide, which further demonstrated that upregulated palmitic acid is important for rescuing the damaged spermatogenesis process by using chestnut polysaccharide and provided an important basis for the follow-up work of this study.

### Palmitic acid could rescue the damaged spermatogenesis process of asthenozoospermia -model mice via enhancing PPARA expression

**Figure 4:**
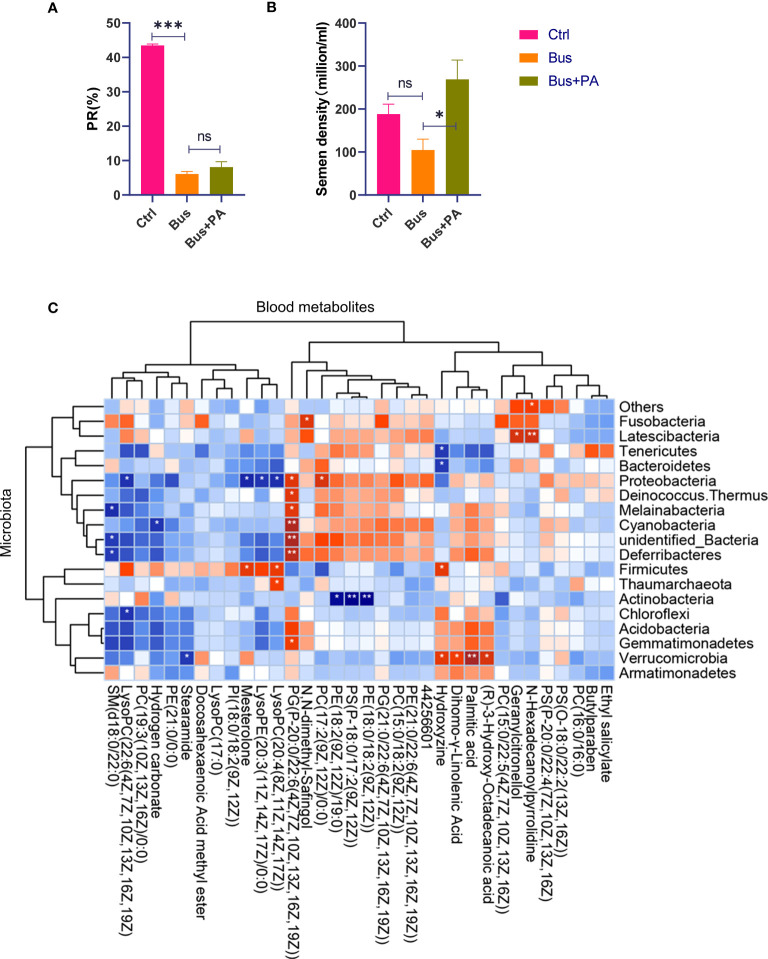
The function verification of palmitic acid on semen quality in vitro and correlation analysis of 16S rDNA and metabolite profiling. **(A)** the statistical analysis of sperm motility from different groups. The asterisk (*) represents a significant difference and the asterisk (***) represents a very significant difference. The abbreviation ns represents a non-significant difference; **(B)** the statistical analysis of semen density from different groups; **(C)** CPs-associated changes in metabolic features and their microbial associations. The asterisk (* or **) represents a significant association. The blue block represents a negative correlation and the red block represents a positive correlation.

After initially determining the important role of palmitic acid upregulation in the rescue of impaired spermatogenesis by chestnut polysaccharide, we further verified the effect of palmitic acid by using asthenozoospermia-model mice, and the results showed that chestnut polysaccharide had the effect of improving semen quality(**Figures 4A, B**), especially on semen density, in addition, the sperm motility of asthenozoospermia-model mice receiving chestnut polysaccharide was also increased to some extent; subsequently, according to the cross comparison analysis between the Swiss Target-Prediction webtool and STITCH database, the key target gene of palmitic acid, *Ppara*, was screened (**Figure 3E**). The protein expression of PPARA in different tissues was also analyzed based on The Human Protein Atlas database, and the results showed that the protein was highly expressed in testis, which also indicated that this protein may play an important role in the physiological function of testis (**Figure 3E**) and this result provided an important theoretical basis for the subsequent use of chestnut polysaccharide in the treatment of asthenozoospermia.

Interestingly, in our association analysis based on previous gut microbiome results (19), we found that only one phylum *Cyanobacteria* was significantly negatively associated with bicarbonate, and most of the remaining bacteria were negatively associated, but not significantly (**Figure 4C**), while only one phylum *Verrucomicrobia* was significantly positively associated with palmitic acid, and most of the remaining bacteria were positively associated, but not significantly, suggesting that *Cyanobacteria* and *Verrucomicrobia* have important roles in maintaining normal spermatogenesis.

## Discussion

In this study, we studied the blood metabolomic changes of busulfan-induced asthenozoospermia-model mice before and after oral administration of chestnut polysaccharide with the help of metabolome, and screened two key differential metabolites (hydrogen carbonate and palmitic acid) from the set of metabolomic changes; we then analyzed the correlation between several metabolites and between different metabolites and intestinal flora by correlation analysis, and found that palmitic acid in the blood serum of mice after oral administration of chestnut polysaccharide had different degrees of correlation with various metabolites, and palmitic acid level had a significant positive correlation with the abundance of *Verrucomicrobia*; finally, we verified the role of palmitic acid in rescuing the damaged spermatogenesis process by using asthenozoospermia-model mice, and confirmed the key target gene for palmitic acid to play the rescuing effect by integrating the analysis of multiple databases. In conclusion, this study found that chestnut polysaccharide rescued the damaged spermatogenesis in asthenozoospermia-model mice by upregulating palmitic acid level, which will provide theoretical basis and technical support for the use of chestnut polysaccharide in the treatment of asthenozoospermia.

It is known that hydrogen carbonate has a crucial role in spermatogenesis (30–33), and the present study further revealed that hydrogen carbonate may be significantly involved in the rescue process mediated by chestnut polysaccharide with the help of metabolomic analysis technique, while further confirming the important role of hydrogen carbonate in spermatogenesis. However, the specific mechanism of action of how chestnut polysaccharide affects hydrogen carbonate is unclear, which will provide us with references and new ideas for our subsequent studies.

The most important point found in this study is that chestnut polysaccharide can significantly increase the expression level of palmitic acid and further improve the impaired spermatogenesis process by increasing the protein expression of PPARA. Previous studies have shown that palmitic acid has an important effect on maintaining semen quality and improving sperm motility (22, 24, 26), and the present study also demonstrated that palmitic acid has a certain effect on salvaging spermatogenic damage with the help of asthenozoospermia-model mice, which also an important theoretical basis for the subsequent in-depth exploration of how palmitic acid enhances the mechanism of action of spermatogenesis through up-regulation of *Ppara* gene expression. In addition, the mechanism of how chestnut polysaccharide increases palmitic acid level and the is still unclear, which will be the direction of our subsequent research.

In conclusion, the present study demonstrated that chestnut polysaccharide significantly altered metabolome of asthenozoospermia-model mice, especially upregulating palmitic acid level, further proving that chestnut polysaccharide has an ameliorative and salvage effect on mice with weak spermatozoa, which will provide theoretical basis and technical support for the use of chestnut polysaccharide in the treatment of asthenozoospermia.

## Data availability statement

The datasets presented in this study can be found in online repositories. The names of the repository/repositories and accession number(s) can be found below: https://ngdc.cncb.ac.cn/gsa, CRA004367. Other data employed in this study are available from the authors upon request.

## Ethics statement

The animal study was reviewed and approved by The Animal Care and Ethics Committee of Qingdao Agricultural University.

## Author contributions

LL and ZS designed the research. BH, JG, and BZ performed the research. CL, TQ, ZM, YJ, and SY generated the data. LH, XZ, and YT analyzed the data. DL and BW provided project oversight. BH, JG, and BZ wrote the paper. All authors contributed to the article and approved the submitted version.
